# A retrospective cohort study: predictive nomogram for clinical pregnancy outcomes after intrauterine insemination

**DOI:** 10.3389/fmed.2026.1912464

**Published:** 2026-09-23

**Authors:** Kunli Feng, Huiqun Yin, Yali Xu, Wan Wang, Yun Zhou

**Affiliations:** 1 Reproductive Medicine Center, Anhui No.2 Provincial People's Hospital, Hefei, China; 2 Anhui Institute of Medicine, Hefei, China

**Keywords:** anti-TPO, clinical pregnancy, intrauterine insemination, nomogram, ovarian stimulation, predictive model

## Abstract

**Introduction:**

Intrauterine insemination (IUI) is a widely used assisted reproductive technique; however, the factors associated with clinical pregnancy remain incompletely understood. This study aimed to identify factors associated with clinical pregnancy and develop an exploratory prediction model for clinical pregnancy after IUI.

**Methods:**

In this retrospective cohort study, we analyzed clinical and laboratory data from 764 IUI cycles performed between January 2019 and December 2024. The data were divided into a training set (75%, 573 cycles) and a validation set (25%, 191 cycles) according to the treatment timing. Multivariable logistic regression analysis was performed to identify factors associated with clinical pregnancy, and a nomogram was developed based on the final model.

**Results:**

In the multivariable logistic regression analysis, female age [odds ratio (OR) = 0.938, 95% confidence interval (CI): 0.885–0.995, *P* = 0.034], body mass index (OR = 1.065, 95% CI: 1.014–1.118, *P* = 0.012), and anti-thyroid peroxidase antibodies (log-transformed, OR = 1.002, 95% CI: 1.000–1.004, *P* = 0.028) were significantly associated with clinical pregnancy. Controlled ovarian stimulation was also associated with clinical pregnancy (OR = 1.843, 95% CI: 1.296–2.623, *P* = 0.048). However, this association should be interpreted cautiously due to potential confounding by indication. Anti-Müllerian hormone was retained in the final model due to its established clinical relevance to ovarian reserve assessment, although it did not reach conventional statistical significance (*P* = 0.051). A nomogram incorporating these five variables was developed, achieving an area under the curve of 0.653 (95% CI: 0.608–0.697).

**Discussion:**

Given its limited discriminative performance, the model should be considered an exploratory tool rather than a definitive clinical decision-making tool. These findings may provide preliminary information for individualized counseling; however, external validation in larger, prospective, multicenter cohorts is required before clinical application.

## 1 Introduction

Intrauterine insemination (IUI) is a technique that involves injecting sperm into a woman's uterine cavity by bypassing sexual intercourse (1). Sperm cells are purified from seminal plasma using sperm washing methods such as swim-up or density gradients (2). IUI is mainly used for patients with mild oligoasthenospermia in men, infertility caused by cervical factors in women, reproductive tract malformations, inability to have sexual intercourse due to psychological factors, and unexplained infertility (3). IUI has the characteristics of simplicity, non-invasiveness, low cost, being closer to natural conception, and no complications associated with oocyte retrieval; however, its clinical pregnancy rate (CPR) has always remained at 8%–25% (4).

Many factors influence the CPR following IUI, including age, infertility type, sperm quality, mature follicular number, and endometrial thickness (5). However, the prognostic value of these factors remains controversial. For example, some studies have shown that double IUI and ovulation induction can increase the pregnancy rate of IUI (6, 7). On the one hand, other studies have argued that the single/double IUI and cycle treatment options do not significantly influence pregnancy rates (8). On the other hand, several studies have indicated that some semen parameters, such as semen volume, sperm motility, and morphology, can predict pregnancy outcomes following IUI (9, 10). However, other studies have shown that these factors have no significant prognostic value (11). In addition, some studies have found that the combination of several influencing factors has an appreciable ability for prognostic classification (12). Therefore, it is also desirable to create a multivariate mathematical model to predict a successful pregnancy following IUI.

## 2 Materials and methods

### 2.1 Study population

A retrospective cohort study was conducted to analyze the clinical data of patients undergoing IUI cycles between January 2019 and December 2024. Prior to enrollment, patients were evaluated to determine the etiology of infertility. Female patients had tubal patency confirmed by hysterosalpingography, and their male partners underwent semen analysis. The indications and contraindications for IUI were determined in accordance with the national clinical guidelines for human-assisted reproductive technology. All pregnancies were confirmed by a positive serum *β*-human chorionic gonadotropin (*β*-hCG) test, performed 14 days after IUI. Ultrasound examinations were repeated 5 weeks after IUI. Demographic data, including age, duration of infertility, routine semen parameters, and IUI pregnancy outcomes, were extracted from patients’ medical records. Cases with missing values for any of the candidate predictor variables were excluded from the analysis. A complete case analysis approach was adopted.

The exclusion criteria were as follows: (1) endometrial thickness ≤5 mm on the day of ovulation; (2) cycle cancelation due to luteinization; and (3) chromosomal abnormalities in either spouse.

### 2.2 Data splitting

Only the first IUI cycle (cycle sequence number = 1) per patient was included, yielding a dataset of 764 cycles from 764 unique women with no repeated measures. The dataset was divided chronologically: the first 573 cycles constituted the training cohort and the subsequent 191 cycles constituted the validation cohort, ensuring complete patient separation between the datasets.

### 2.3 Follicular monitoring and treatment plan

Transvaginal ultrasonography was performed to monitor follicular and endometrial development. Natural cycle treatment was administered to patients with regular menstrual cycles or those who had previously shown dominant follicle development and ovulation in natural cycles. Controlled ovarian stimulation (COS) was administered to patients with infrequent menstruation, poor follicular development in natural cycles, ovulation disorders, or those who had previously failed to conceive with IUI in natural cycles.

The ovulation induction protocol included letrozole, human menopausal gonadotropin (hMG), or letrozole combined with hMG. Medication was adjusted individually based on follicular and endometrial growth monitored by ultrasound, aiming to control the number of dominant follicles to fewer than three. Once the dominant follicle reached a diameter of 16–18 mm, hCG (10,000 U) or recombinant hCG (250 μg, subcutaneous injection) was administered based on the levels of luteinizing hormone (LH), estradiol, and progesterone.

### 2.4 Sperm preparation and IUI

The male partner abstained from ejaculation for 2–7 days before the procedure. On the day of IUI, semen was collected by masturbation. After liquefaction for 30 min, the semen was processed using a double-layer density gradient centrifugation method, and 0.5–0.6 mL of sperm suspension was collected for insemination. Follicular monitoring began on days 10–11 of the patient's menstrual cycle. When the follicle diameter reached ≥16 mm, the follicle size, LH, and estradiol levels were monitored daily. Insemination was performed when LH levels exceeded 2–3 times the baseline value. The processed sperm suspension (0.5–0.6 mL) was slowly injected into the uterine cavity through a standard insemination catheter that was inserted approximately 1 cm above the internal cervical os. The patient maintained a supine position for approximately 15 min after the procedure. The single IUI group underwent one insemination per cycle, while the double IUI group underwent two inseminations within the same cycle (one before ovulation and one after ovulation). In this study, TPMSC refers to the total progressive motile sperm count in the post-wash insemination samples. It was calculated as follows:

$$\mathrm{TPMSC}=\mathrm{semen}\;\mathrm{volume}\;(\mathrm{mL})\times \mathrm{sperm}\;\mathrm{concentration}\;(10^{6}/\mathrm{mL})\times \mathrm{progressive}\;\mathrm{motility}\;(\text{\%})/100.$$

TPMSC was expressed in units of ×10^6^ and measured immediately after semen preparation and before insemination.

### 2.5 Luteal support

After ovulation, 10 mg dydrogesterone was administered orally twice daily for luteal support. On day 14 post-insemination, a serum hCG pregnancy test was performed. Serum hCG levels >10 U/L indicated a biochemical pregnancy, and luteal support therapy was continued. Luteal support therapy was discontinued if pregnancy did not occur. A transvaginal ultrasound was performed on days 28 and 35 post-procedure. The presence of a gestational sac or fetal heartbeat confirmed a clinical pregnancy.

### 2.6 Statistical analysis

Statistical analyses were performed using SPSS Statistics, Python, and R software. Normally distributed data are expressed as mean ± standard deviation (x̅ ± s), and independent samples *t*-tests were used for comparisons between groups. Count data were expressed as rates or proportions (%), and chi-square tests or Fisher's exact tests were used for comparisons between groups. Standardized mean differences (SMD) were calculated to assess the baseline balance between the training and validation cohorts; an SMD <0.1 was considered indicative of a good balance. All continuous variables, including female age, body mass index (BMI), and anti-Müllerian hormone (AMH) levels, were entered into the regression analyses as continuous variables. Anti-thyroid peroxidase (anti-TPO) antibody titers were log-transformed [log(anti-TPO + 1)] prior to regression analysis because of the highly skewed distribution of values (range: 0.2–1,308 IU/mL). The receiver operating characteristic (ROC) curve was used to evaluate the predictive performance of the nomogram, calibration curves were used to assess the distribution bias of the model's predicted values, and decision curve analysis (DCA) and clinical impact curve (CIC) were used to evaluate the model's clinical utility. This study was reported following the Strengthening the Reporting of Observational Studies in Epidemiology (STROBE) guidelines and the Transparent Reporting of a Multivariable Prediction Model for Individual Prognosis or Diagnosis (TRIPOD) statement.

No missing values were identified for any of the five predictor variables. Clinical pregnancy outcomes were recorded as 1 (pregnant) or 0 (non-pregnant). Therefore, a complete case analysis approach was adopted.

### 2.7 Variable selection and model development

Candidate predictors were selected *a priori* based on their clinical relevance and availability in routine practice. Variables with *P* < 0.05 in univariate screening were entered into the multivariable Firth logistic regression. AMH was additionally retained, regardless of statistical significance, based on its established biological relevance to the ovarian reserve and its inclusion in prior IUI prediction models. The final model used to construct the nomogram was as follows: logit[P(clinical pregnancy)] = *β*0 + *β*1(female age) + *β*2(BMI) + *β*3(AMH) + *β*4([anti-TPO log-transformed]) + *β*5(COS). COS was coded as (0 = reference treatment; 1 = COS), and continuous variables were modeled as linear continuous variables. Due to the limited number of early miscarriage events (*n* = 24, events per variable [EPV] < 10), multivariable analysis was not performed; instead, descriptive statistics and univariate comparisons among clinical pregnancies (*n* = 186) are presented. Multicollinearity was assessed using the variance inflation factor (VIF); all retained variables had a VIF of <1.2, indicating no significant collinearity.

## 3 Results

### 3.1 IUI statistical analysis flowchart

The IUI flowchart is shown in Figure 1.

**Figure 1:**
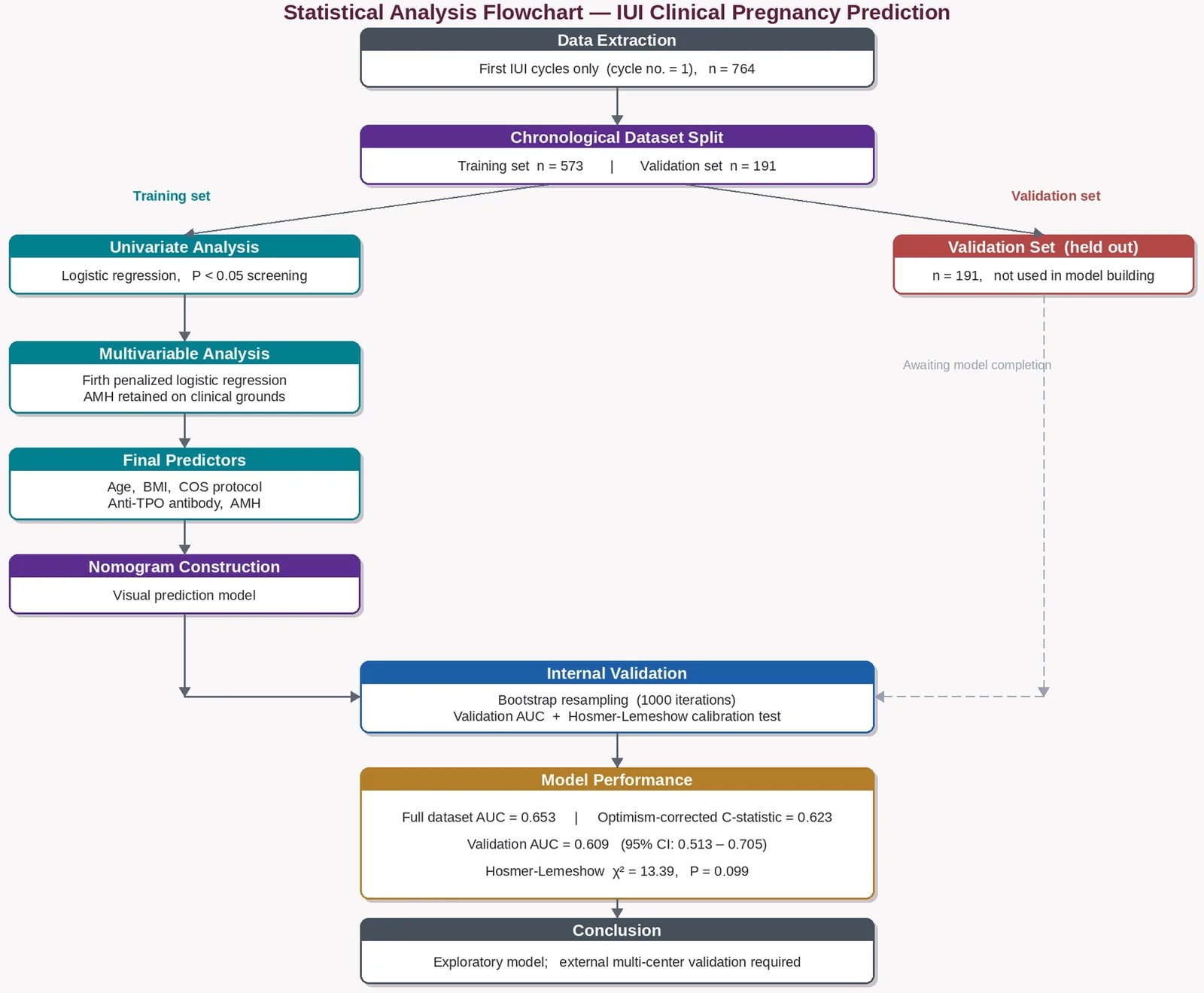
Statistical analysis workflow for the development and validation of a clinical pregnancy prediction model for intrauterine insemination.

### 3.2 General condition of the patient and cycle outcomes

A total of 764 IUI cycles were analyzed, of which 186 resulted in clinical pregnancy, with a CPR of 24.35% (186/764), an early miscarriage rate (EMR) of 12.90% (24/186), and a live birth rate (LBR) of 18.1% (138/764). Of the 186 clinical pregnancies, 138 resulted in live births, 4 ended in early miscarriage (<12 weeks), 8 in ectopic pregnancy, 12 in late miscarriage (≥12 weeks), and 4 were lost to follow-up/outcome pending at the study cutoff. The cohorts were divided into a training set (75%, 573 IUI cycles) and a validation set (25%, 191 IUI cycles) based on the treatment duration. The training and validation cohorts were balanced across most of the baseline characteristics. However, statistically significant differences were observed in AMH, anti-thyroglobulin antibody (anti-TG), and the proportion of single versus double IUI procedures (SMD >0.2) (Table 1).

**Table 1:** Baseline characteristics of IUI cycles in the training and validation sets.

Items	Validation set	Training set	t/*χ*^2^ value	*P*-value	SMD
No. of cycles	191	573			
Type of infertility			1.284	0.257	0.096
Primary	76.44 (146/191)	72.25 (414/573)			
Secondary	23.56 (45/191)	27.75 (159/573)			
Infertility duration (year)	2.43 ± 1.06	2.61 ± 2.02	−1.214	0.225	−0.012
Female age (year)	29.74 ± 4.65	30.33 ± 3.95	−1.707	0.088	−0.137
Male age (year)	31.49 ± 5.89	31.60 ± 4.58	−0.266	0.790	−0.021
Female BMI (kg/m^2^)	22.68 ± 3.20	23.18 ± 3.63	−1.717	0.086	−0.146
FSH	6.49 ± 1.16	6.82 ± 2.37	−1.935	0.053	−0.177
LH	6.40 ± 3.73	6.83 ± 4.46	−1.189	0.235	−0.105
FSH/LH	1.26 ± 0.63	1.51 ± 3.28	−1.040	0.299	−0.106
AMH (ng/mL)	4.48 ± 2.76	6.17 ± 4.39	−4.998	<0.001	−0.461
Anti-TPO	158.88 ± 341.14	169.19 ± 322.23	−0.377	0.706	−0.031
Anti-TG	7.27 ± 18.98	32.28 ± 100.63	−3.413	<0.001	−0.345
Endometrial thickness on hCG day	7.08 ± 2.72	7.10 ± 2.88	−0.090	0.929	−0.007
Antral follicle count	19.98 ± 5.91	19.34 ± 7.81	0.772	0.440	0.069
Number of IUI cycles			2.969	0.563	0.158
1	57.07 (109/191)	61.08 (350/573)			
2	29.84 (57/191)	27.57 (158/573)			
3	10.47 (20/191)	9.77 (46/573)			
4	2.62 (5/191)	2.62 (15/573)			
5	0 (0/191)	0.70 (4/573)			
Cycle treatment options			0.070	0.791	0.022
Natural cycle	50.26 (96/191)	50.79 (291/573)			
Ovulation cycle	49.74 (95/191)	49.21 (282/573)			
Single/double IUI			18.654	<0.001	−0.390
Single	13.61 (26/191)	29.32 (168/573)			
Double	86.39 (165/191)	70.68 (405/573)			
TPMSC	83.78 ± 12.60	84.92 ± 12.38	−1.100	0.272	−0.091
Clinical pregnancy rate (%)	22.51 (43/191)	24.96 (143/573)	0.464	0.496	−0.057
Early miscarriage rate (%)	9.30 (4/43)	13.99 (20/143)	0.645	0.422	−0.146
Live birth rate (%)	74.42 (32/43)	74.13 (106/143)	0.190	0.663	0.077

Continuous variables are presented as mean ± standard deviation (SD).

SMD, standardized mean difference; IUI, intrauterine insemination; BMI, body mass index; FSH, follicle-stimulating hormone; LH, luteinizing hormone; AMH, anti-Müllerian hormone; anti-TPO, anti-thyroid peroxidase antibodies; anti-TG, anti-thyroglobulin antibodies; hCG, human chorionic gonadotropin; TPMSC, total progressive motile sperm count.

The number in parentheses indicates the number of positive/total; SMD <0.1 indicates good balance; 0.1–0.2 indicates mild imbalance; >0.2 indicates meaningful imbalance between cohorts.

### 3.3 Univariate analysis of factors affecting pregnancy outcomes in IUI

In the univariate analyses, male age, female age, female BMI, AMH, anti-TPO, anti-TG, and cycle treatment protocol were significantly associated with CPR (all *P* < 0.05). Female BMI, endometrial thickness on the day of hCG administration, and progressive sperm motility before sperm processing were significantly associated with EMR (all *P* < 0.05) (Table 2).

**Table 2:** Univariate analysis of factors associated with clinical pregnancy outcomes in IUI cycles.

Items	Clinical pregnancy		Early miscarriage	
	OR (95% CI)	*P*-value	OR (95% CI)	*P*-value
Type of infertility				
Primary	1		1	
Secondary	1.299 (0.903–1.868)	0.158	1.181 (0.481–2.901)	0.716
Infertility duration (year)	1.038 (0.951–1.133)	0.402	0.973 (0.770–1.229)	0.819
Female age (year)	0.951 (0.911–0.992)	**0** **.** **019**	1.075 (0.954–1.210)	0.235
Male age (year)	0.952 (0.917–0.988)	**0** **.** **010**	1.006 (0.907–1.116)	0.913
Female BMI (kg/m^2^)	1.077 (1.028–1.127)	**0** **.** **002**	1.168 (1.044–1.307)	**0** **.** **007**
FSH	0.986 (0.749–1.298)	0.920	1.016 (0.814–1.267)	0.891
LH	1.001 (0.964–1.040)	0.949	0.906 (0.778–1.054)	0.202
FSH/LH	0.806 (0.407–1.596)	0.536	1.606 (0.923–2.792)	0.094
AMH (ng/mL)	0.890 (0.814–0.972)	**0** **.** **009**	1.032 (0.945–1.128)	0.484
Anti-TPO	1.002 (1.000–1.003)	**0** **.** **039**	0.793 (0.599–1.051)	0.107
Anti-TG	1.003 (1.001–1.006)	**0** **.** **017**	1.001 (0.999–1.004)	0.334
Endometrial thickness on hCG day	0.993 (0.936–1.052)	0.805	0.828 (0.693–0.988)	**0** **.** **037**
Total follicle counts	1.016 (0.994–1.038)	0.164	1.069 (0.991–1.153)	0.083
Number of IUI cycles		0.788		0.934
1	1		1	
2	0.867 (0.592–1.268)	0.461	0.710 (0.253–1.988)	0.514
3	1.031 (0.572–1.855)	0.920	0.926 (0.214–4.013)	0.918
Cycle treatment options				
Natural cycle	1		1	
Ovulation cycle	1.575 (1.091–2.273)	**0** **.** **015**	1.331 (0.482–3.677)	0.581
Single/double IUI				
Single	1		1	
Double	0.836 (0.577–1.213)	0.346	0.729 (0.295–1.799)	0.493
Sperm concentration before processing	1.002 (0.998–1.005)	0.366	1.003 (0.995–1.011)	0.411
Sperm viability before processing (%)	0.991 (0.980–1.001)	0.091	0.975 (0.949–1.002)	0.071
PR before processing (%)	0.991 (0.981–1.002)	0.114	0.970 (0.942–0.999)	**0** **.** **045**
PR after processing (%)	1.008 (0.994–1.022)	0.284	0.974 (0.943–1.007)	0.119
TPMSC	1.000 (0.993–1.007)	0.967	0.999 (0.983–1.016)	0.937

CI, confidence interval; BMI, body mass index; FSH, follicle-stimulating hormone; LH, luteinizing hormone; AMH, Anti-Müllerian hormone; anti-TPO, anti-thyroid peroxidase antibodies; anti-TG, anti-thyroglobulin antibodies; hCG, human chorionic gonadotropin; IUI, intrauterine insemination; PR, progressive motility before processing; TPMSC, total progressive motile sperm count. Anti-TPO values were log-transformed prior to analysis. OR represents change per one log-unit increase in anti-TPO. *P*-values <0.05 are in bold.

### 3.4 Multivariate logistic regression analysis of factors affecting pregnancy outcomes

In the multivariable logistic regression analysis, female age, BMI, and anti-TPO remained significantly associated with CPR (*P* < 0.05). Ovulation induction treatment was also associated with higher odds of clinical pregnancy than the natural cycle. However, given the potential for confounding by indication, this association should not be interpreted as a causal effect of the treatment. After adjusting for other covariates, male age and anti-TG were no longer significantly associated with CPR; AMH was retained *a priori* based on its established biological relevance to ovarian reserve assessment, independent of statistical significance [Wald *P* = 0.051; likelihood ratio test (LRT), *P* = 0.662] (Table 3).

**Table 3:** Multivariable model including all univariately significant predictors.

Items	Clinical pregnancy	
	OR (95% CI)	*P*-value
Female age (years)	0.938 (0.885–0.995)	**0** **.** **034**
Male age (years)	0.995 (0.930–1.063)	0.874
Female BMI (kg/m^2^)	1.065 (1.014–1.118)	**0** **.** **012**
AMH (ng/mL)	0.800 (0.640–1.001)	**0** **.** **051**
Anti-TPO	1.002 (1.000–1.004)	**0** **.** **028**
Anti-TG	0.917 (0.833–1.009)	0.075
Cycle treatment options		
Natural cycle	1	
Ovulation cycle	1.843 (1.296–2.623)	**0** **.** **048**

CI, confidence interval; BMI, body mass index; AMH, anti-Müllerian hormone; anti-TPO, anti-thyroid peroxidase antibodies; anti-TG, anti-thyroglobulin antibodies. Anti-TPO values were log-transformed prior to analysis. OR represents change per one log-unit increase in anti-TPO. *P*-values <0.05 are in bold.

### 3.5 Development and validation of the nomogram prediction model

Based on the final multivariable logistic regression model, a nomogram was developed incorporating female age, BMI, AMH, anti-TPO, and treatment regimen as predictors of CPR (Figure 2A). The discriminative performance of the nomogram was evaluated using ROC curve analysis. The nomogram yielded an area under the curve (AUC) of 0.653 [95% confidence interval (CI): 0.608–0.697] (Figure 2B), indicating a limited discriminative ability. The calibration curve of the training set showed the relationship between the predicted and observed probabilities, with the bias-corrected curve generally following the diagonal reference line (Figure 2C).

**Figure 2:**
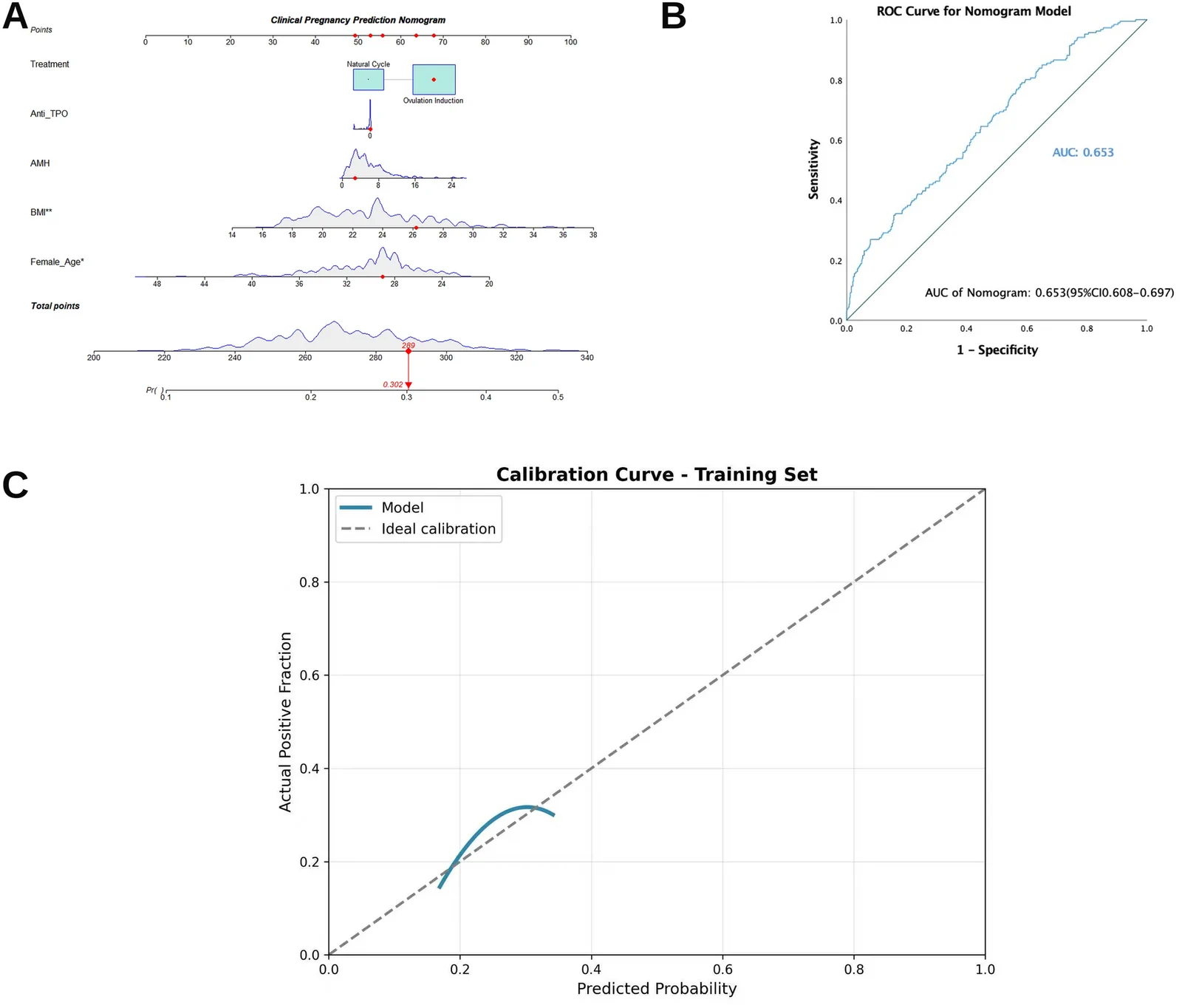
Development and internal validation of the CPR prediction model. **(A)** The nomogram exhibited five characteristics of a patient (treatment = ovulation induction, anti-TPO = 40.3, AMH = 2.8, BMI = 26.2, female age = 29, with a total score of 289 points, and the predicted probability of CPR was 30.2%). **(B)** The AUC of the nomogram model was 0.653 (95% CI: 0.608–0.697). **(C)** Calibration curve of the training set.

### 3.6 Bootstrap validation and cross-validation

Bootstrap internal validation (*B* = 1,000 resamples) of the full dataset yielded an apparent AUC of 0.653, an optimism of 0.030, and an optimism-corrected C-statistic of 0.623 (Figure 3A). The repeated 10-fold cross-validation yielded a mean AUC of 0.624 ± 0.071 (Figure 3B).

**Figure 3:**
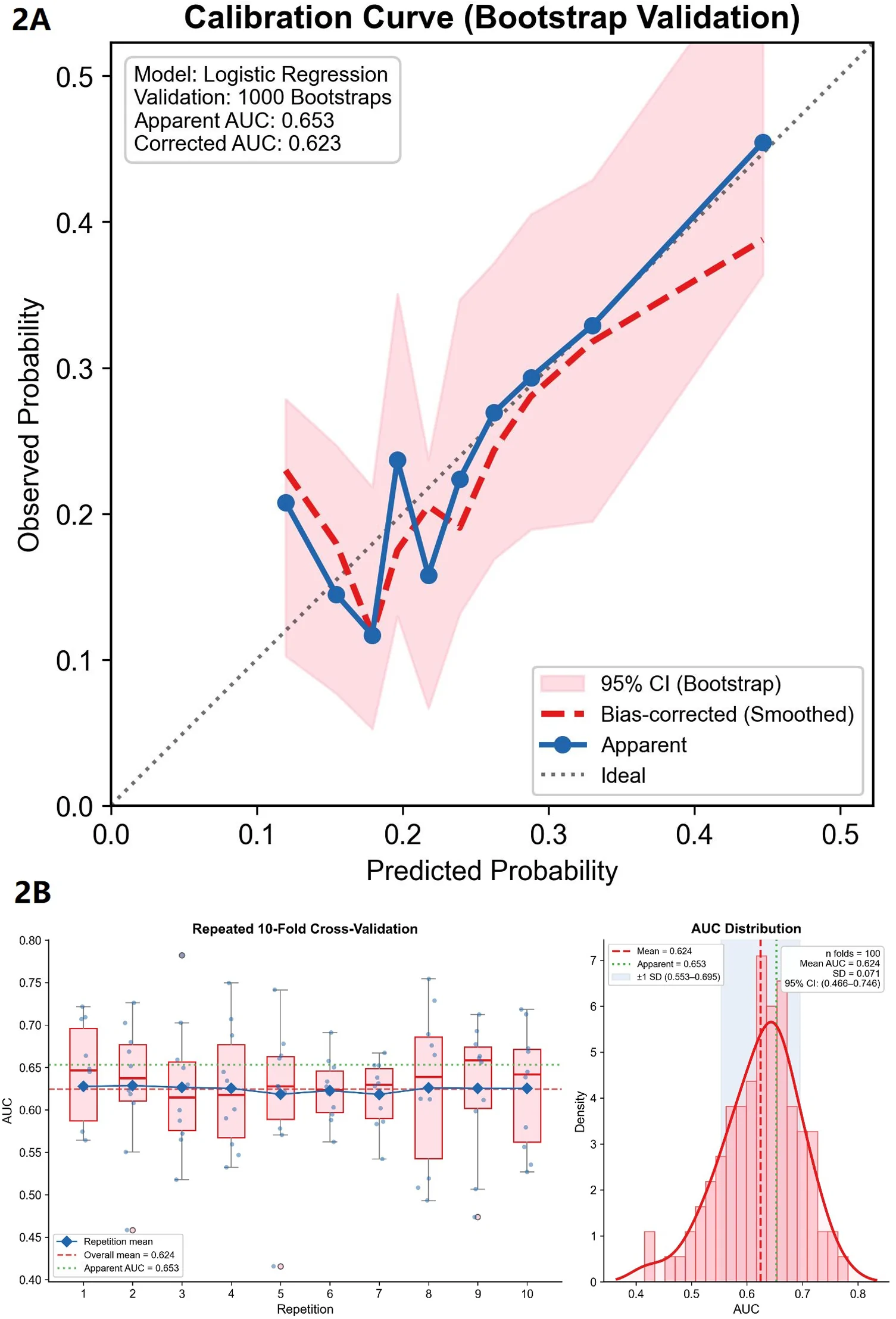
Bootstrap and cross-validation. Internal validation of the prediction model. **(A)** Bootstrap calibration curve (*n* = 1,000; corrected AUC = 0.623). **(B)** Repeated 10-fold cross-validation AUC distribution (mean = 0.624).

### 3.7 Discrimination and calibration of the nomogram in the temporal validation cohort

The discriminative ability in the temporal validation cohort (*n* = 191) was an AUC of 0.609 (95% CI: 0.513–0.705) (Figure 4A). The calibration curve shows the agreement between the nomogram-predicted and observed clinical pregnancy rates. The Hosmer–Lemeshow (HL) goodness-of-fit test indicated acceptable calibration (*χ*^2^ = 13.39, *P* = 0.099) (Figure 4B).

**Figure 4:**
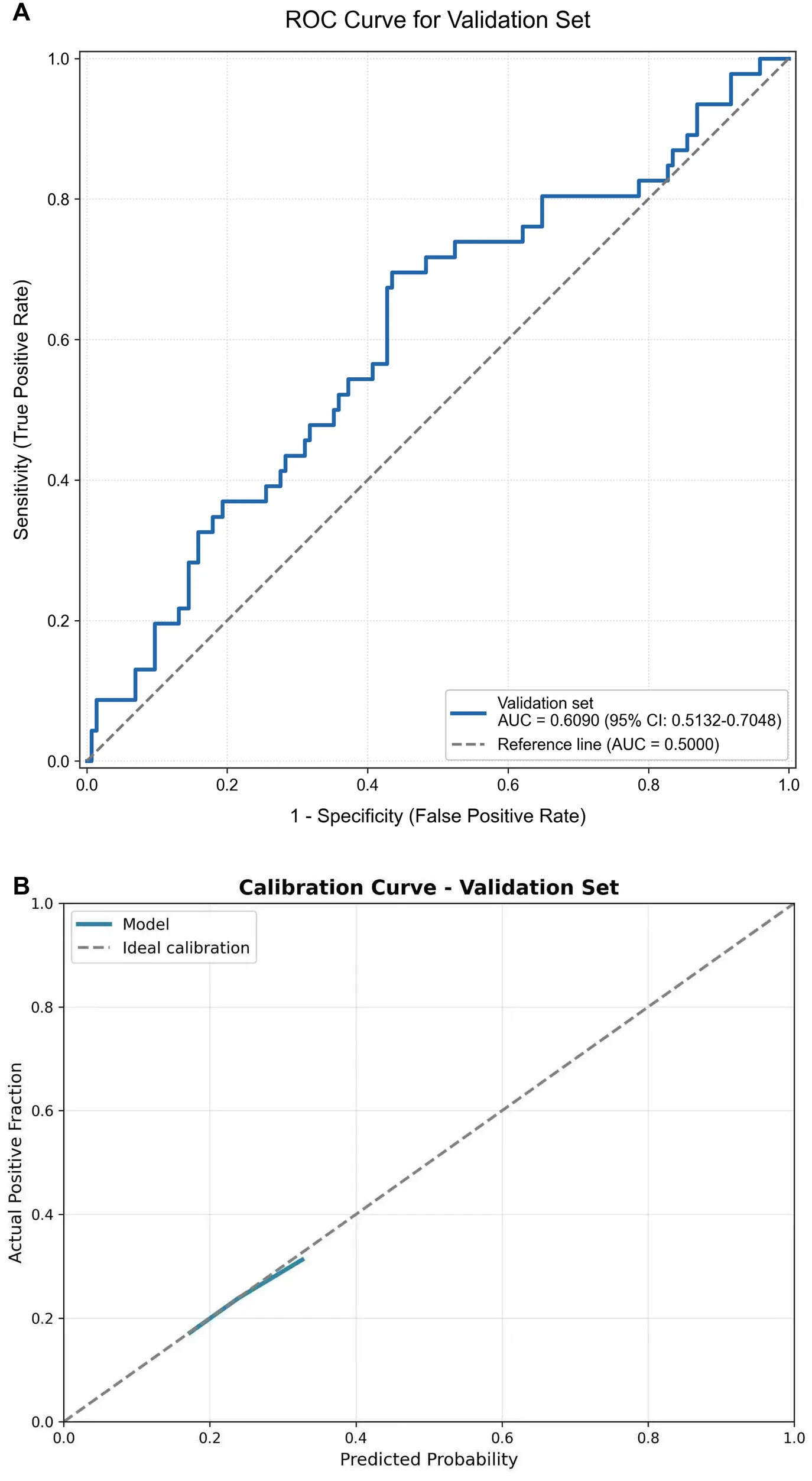
Development and internal validation of the CPR prediction model. **(A)** The AUC of the validation set was 0.609 (95% CI: 0.513–0.705). **(B)** Calibration curve of the validation set.

### 3.8 Clinical utility of the prediction model

DCA demonstrated a net clinical benefit primarily at threshold probabilities of 0.10–0.30, consistent with the prevalence of clinical pregnancy in this IUI cohort. At threshold probabilities above 0.30, the net benefit was minimal, reflecting the limited discriminative ability of the model in higher-risk strata (Figure 5). In the CIC analysis, the apparent consistency above a threshold of 0.50 should be interpreted cautiously, as few patients were classified as high-risk at these thresholds (Figure 6).

**Figure 5:**
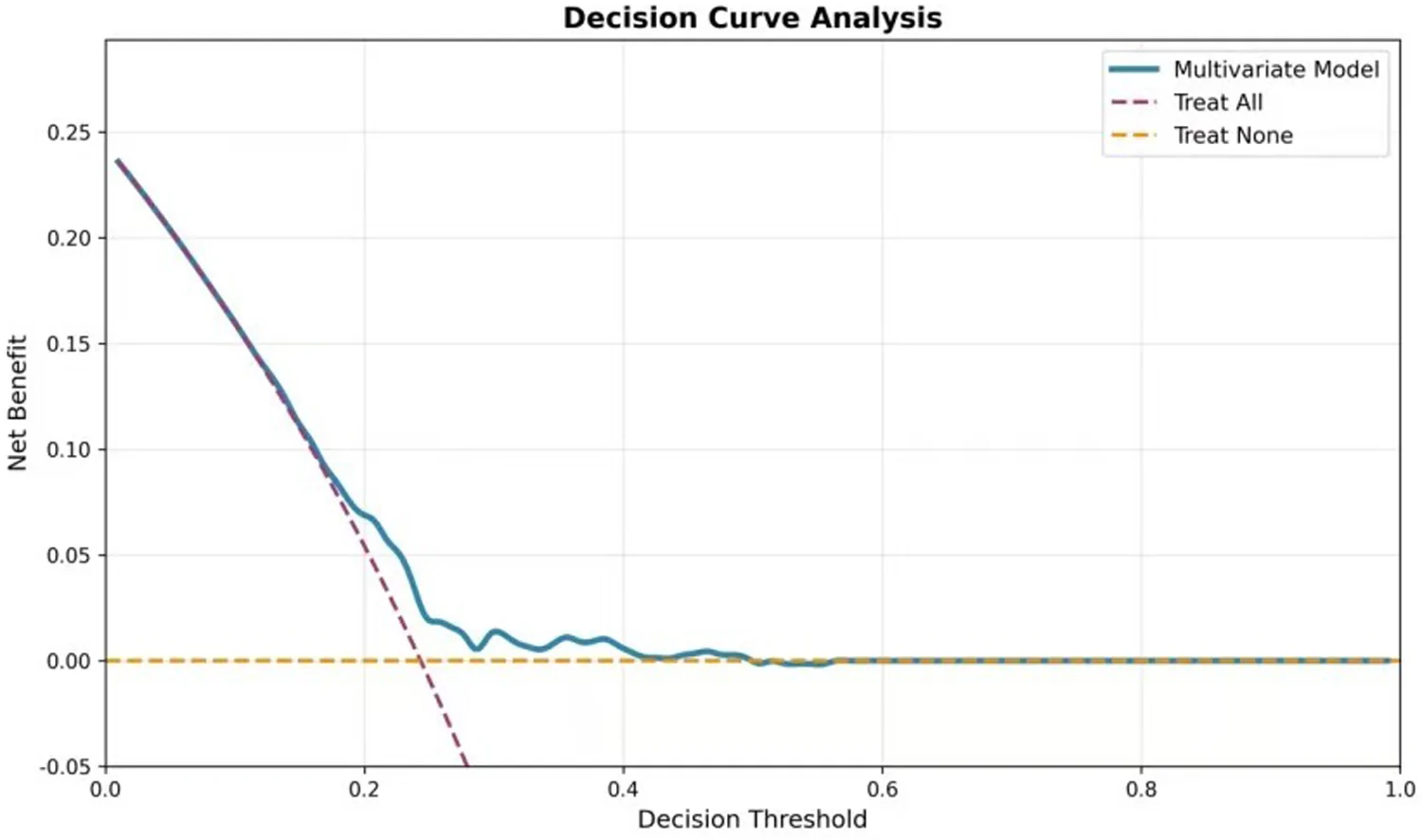
Decision curve analysis of the prediction model. The “multivariate model” curve (blue) shows the net benefit of using the nomogram for decision-making. The “treat all” curve (red) represents net benefit if all patients were treated, while the “treat none” curve (yellow) represents the net benefit if no patients were treated.

**Figure 6:**
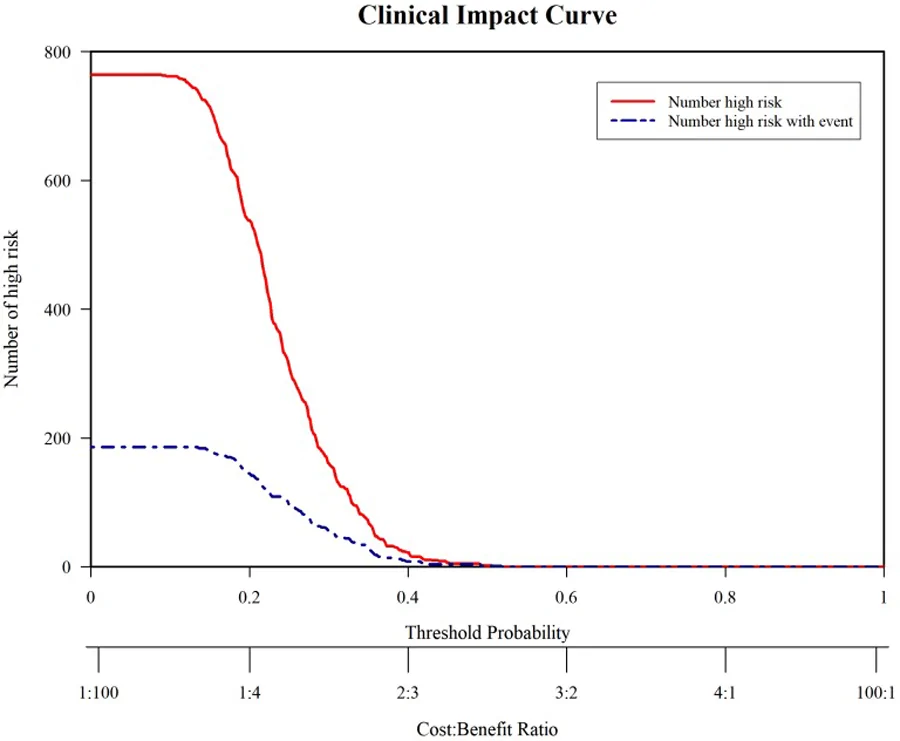
Clinical impact curve of the prediction model. Red line: number of patients classified as high-risk at each threshold probability. Blue line: number of high-risk patients who actually achieved clinical pregnancy at each threshold.

## 4 Discussion

In recent years, the incidence of infertility has steadily increased worldwide. Data from the national reproductive health epidemiological survey published by Qiao et al. showed that the incidence of infertility in China has continuously increased over the past 13 years, from 12% in 2007 to 18% in 2020 (13). In 2018, the European Society for Human Reproduction and Embryology reported that 20%–30% of infertility cases were attributed to male causes, 20%–35% to female causes, and 25%–40% to both (14). IUI is a simple and widely used assisted reproductive technology suitable for mild male factor infertility, ovulation disorders, mild endometriosis, and unexplained infertility. Previous studies have shown that many factors can affect the pregnancy outcome of IUI, including the patient's baseline condition, the quality of the male's semen, the type of infertility, and the stimulation protocol (14). Some studies have also indicated an association between thyroid autoimmunity and adverse pregnancy outcomes; however, the mechanism is not yet fully understood (15). Our center strictly adheres to relevant clinical guidelines when performing IUI treatment. For patients whose semen parameters do not meet the standards, we generally recommend abandoning IUI or switching to *in vitro* fertilization and embryo transfer (IVF-ET). Therefore, among couples with semen parameters meeting the predefined criteria, female age, BMI, AMH, anti-TPO, and cycle treatment regimen were identified as factors associated with clinical pregnancy. Based on these variables, we developed a nomogram to estimate the probability of clinical pregnancy after IUI.

As women age, their ovarian reserve decreases, endometrial receptivity declines, and systemic diseases may develop. As an effective indicator of ovarian reserve, AMH levels continuously decline with age, while oocyte quality decreases and aneuploidy increases, leading to a decline in natural fertility (16). Although not all studies indicate differences in CPR when stratified by age, previous studies have shown a decreasing trend in CPR with increasing age (17–19). A randomized trial comparing CPR in natural cycles and ovulation induction cycles for couples with unexplained or male factor infertility showed that age was the only prognostic indicator, with women aged 38 years having a 50% lower chance of conception compared to women aged 28 years (19). In this study, after adjusting for other confounding factors in the logistic regression equation, age remained an independent factor influencing CPR. The LRT confirmed that female age contributed significantly to the model fit (*χ*^2^ = 7.839, *P* = 0.005). The marginal contribution of AMH was not significant by LRT (*χ*^2^ = 0.192, *P* = 0.662) and had a Wald *P* of 0.051 in the multivariable model; it was retained *a priori* based on its established biological relevance to the ovarian reserve assessment. Higher AMH levels were associated with lower odds of clinical pregnancy in our cohort (OR = 0.80). This inverse association may be related to potential confounding factors such as polycystic ovary syndrome (PCOS) or ovulatory dysfunction, which can be associated with elevated AMH levels. However, given the retrospective design and lack of systematically collected information on PCOS and related clinical factors, residual confounding cannot be excluded. Therefore, this finding should be regarded as hypothesis-generating and requires validation in future prospective studies.

Obesity has become a global pandemic. According to the World Health Organization (WHO) recommendations, the normal adult BMI is between 18.5 and 23.9 kg/m^2^. Studies have shown that BMI is an important predictive factor for CPR, and there is a tendency toward a decrease in reproductive outcomes (number of inseminations with positive human chorionic gonadotropin, number of live births, and number of term births) with an increase in BMI (20, 21). Abnormal BMI values can lead to endocrine disorders, metabolic abnormalities, decreased ovarian responsiveness, and endometrial receptivity, which in turn can cause abnormal follicle development, ovulation, and embryo implantation problems. However, a 2023 meta-analysis and systematic review showed that female BMI was not significantly associated with pregnancy outcomes post-IUI (22, 23). Meanwhile, the value of obesity as a predictor of infertility treatment outcomes remains controversial. In our study, BMI remained significantly associated with clinical pregnancy after adjusting for potential confounding factors.

Existing research has demonstrated that factors such as endometrial thickness on the day of hCG insemination, the total number of progressively motile sperm after insemination, and the number of IUI attempts are significantly correlated with pregnancy outcomes after IUI (24). Our study showed that women's BMI, endometrial thickness on the day of hCG administration, and the total number of progressively motile sperm after semen processing were associated with EMR. Owing to the limited number of early miscarriage events (*n* = 24, EPV < 10), multivariable analysis was not performed. Appropriate endometrial thickness (8–15 mm) and a structurally stable endometrium are important for successful pregnancy after IUI (25). An endometrium that is too thin (<7 mm) can lead to embryo implantation failure, and even if pregnancy is successful, recurrent miscarriages are likely to occur (26). When the endometrium is too thin, clinical intervention should be implemented promptly to improve adverse pregnancy outcomes after IUI. In IUI treatment, there is still no definitive conclusion regarding the specific minimum suitable sperm count. The literature reports that with an increase in sperm density after treatment, the CPR of IUI increases, but the difference is not statistically significant (12). The results of this study support this hypothesis. Therefore, if the only cause of infertility is male asthenospermia, IUI treatment should be prioritized.

There is an association between thyroid autoimmunity and poor pregnancy outcomes in IUI, but the underlying mechanism is not fully understood. The prevalence of autoimmune thyroid disease is 5%–20% in women of reproductive age but reaches 25% in infertile women (27, 28). Studies have confirmed the presence of thyroid autoantibodies in the follicular fluid of infertile women with autoimmune thyroid disease but normal thyroid function (29). The clinical features include anti-TPO and anti-TG, and it is speculated that thyroid autoantibodies have a direct effect on the reduced fertilization rate of oocytes and the reduced rate of high-quality embryos in these patients (28). Anti-TPO and anti-TG were included in the univariate analysis and baseline characteristics table as clinically relevant thyroid autoimmune markers. However, anti-TG did not reach statistical significance in the multivariate logistic regression analysis (*P* = 0.075). Anti-TPO antibody titers were log-transformed prior to regression analysis because of the highly skewed distribution of values in our cohort (range: 0.2–1,308 IU/mL). Nonetheless, the effect size should be interpreted cautiously, and it should be acknowledged that our dataset did not systematically capture thyroid function test results or treatment history, which limits the interpretability of this association, a clinically meaningful effect, given that markedly elevated anti-TPO is not uncommon in women with thyroid autoimmunity. These findings provide preliminary evidence that thyroid autoimmunity may contribute to IUI outcomes. The potential value of anti-TPO assessment in pre-IUI evaluation warrants further investigation in larger, prospective, multicenter studies before formal clinical recommendations can be made.

The effect of treatment protocols on pregnancy outcomes after IUI remains controversial. Some studies suggest that there is no statistically significant difference in pregnancy rates between natural and stimulated cycles, with natural cycles sometimes showing a higher rate (30, 31). Other studies suggest that the pregnancy rate in stimulated cycles is higher than that in natural cycles (1, 32). A meta-analysis by van Rumste et al. (33) showed that ovulation induction protocols can promote the development of multiple follicles, thereby increasing CPR. The present study also found an association between stimulation cycles and a higher CPR compared with that of natural cycles. The observed association between the COS protocol and CPR should be interpreted with caution, as COS was assigned based on clinical indications, introducing potential confounding by indication. The associations of anti-TPO and AMH with CPR may similarly be influenced by unmeasured confounders, including thyroid function status, levothyroxine treatment, PCOS diagnosis, and ovulatory dysfunction, which were not systematically recorded in this study. Nevertheless, considering the risks of ovarian hyperstimulation and multiple pregnancies, ovulation induction should be applied with caution. For patients with regular menstrual cycles and normal ovulation, we suggest using a natural cycle protocol for the first two cycles. It should be noted that the COS variable was coded as binary (stimulated vs. natural cycle) and did not capture heterogeneity among different stimulation protocols, which may represent a source of residual confounding.

In our study, the clinical pregnancy rate was 24.35%. This rate may be influenced by differences in patient characteristics, treatment protocols, and clinical practice patterns across IUI populations. Therefore, comparisons with pregnancy rates reported in other studies should be interpreted with caution. Notably, no multiple pregnancies were observed in our cohort, which may reflect the clinical management strategies used in our center.

The dataset was divided chronologically rather than randomly to reflect real-world clinical practice and avoid data leakage that may arise from the random splitting of temporally autocorrelated clinical data. This approach simulates prospective model application, where the model trained on earlier cases is applied to subsequently treated patients, thereby providing a more realistic assessment of predictive performance.

The nomogram achieved an AUC of 0.653 (95% CI: 0.608–0.697), reflecting a moderate discriminative ability. This level of AUC performance is consistent with previously published IUI prediction models: the Mayo Clinic nomogram reported an optimism-corrected C-statistic of 0.611 (34), and a machine-learning model in a large Chinese population achieved an AUC of 0.716 (35), suggesting that the biological complexity of IUI outcomes limits achievable prediction accuracy across methodologies (36).

## 5 Limitations

1. The model should not be generalized to women with a very thin endometrium or to centers that perform IUI under different endometrial criteria.
2. The moderate discriminative ability of the model (AUC = 0.653) limits its utility for binary decision-making at the individual level. It should be regarded as a supplementary exploratory tool, pending external validation. Future external multicenter validation is warranted.
3. Variable selection based on univariate pre-screening (*P* < 0.05) is a standard but imperfect approach; future studies should consider least absolute shrinkage and selection operator (LASSO) regression or pre-specified candidate variable sets to reduce the selection bias.
4. Confounding by indication for COS and unmeasured thyroid/metabolic confounders for anti-TPO and AMH levels represent important limitations of the present analysis.

## 6 Conclusion

The developed nomogram demonstrated limited discriminative ability (optimism-corrected C-statistic: 0.623; validation AUC: 0.609; 95% CI: 0.513–0.705). These findings should be regarded as exploratory and hypothesis-generating. The model requires independent external validation in multicenter cohorts before consideration for clinical application. Given this level of performance, the nomogram is not intended to replace clinical judgment or serve as a definitive decision-making tool, but may provide supplementary probabilistic information to support individualized pre-treatment counseling and assist clinicians in identifying patients who may benefit from alternative treatment strategies.

## Data Availability

The original contributions presented in this study are included in the article/Supplementary Material. Further inquiries can be directed to the corresponding author/s.
